# Signal relay in *C. elegans*: A tissue-perspective on coordinating organismal proteostasis and its impact on aging

**DOI:** 10.1016/j.cstres.2025.100127

**Published:** 2025-10-20

**Authors:** Loren Cocciolone, Akhil Souparnika, Valeria Uvarova, Katie Kessler, Patricija van Oosten-Hawle

**Affiliations:** Department of Biological Sciences, University of North Carolina at Charlotte, Charlotte, NC 28223

**Keywords:** Organismal proteostasis, Aging, Proteotoxicity, Neurodegenerative diseases, Transcellular stress signaling

## Abstract

As the global demographics shifts towards an increasingly aging population, understanding the effects and molecular mechanisms underlying aging becomes more and more important within biomedical research. A hallmark of aging is the progressive deterioration of protein homeostasis (proteostasis), characterized by the accumulation of misfolded protein aggregates within the cell. The proteostasis network is essential in mitigating the harmful effects of proteotoxic aggregates by activating stress response and degradation pathways. Significant discoveries in aging research are often inherently intertwined with proteostasis, many of which were made using the invertebrate *Caenorhabditis elegans*. Many longevity pathways, such as the insulin-like signaling pathway, initially identified in *C. elegans,* are mediated through inter-tissue stress signaling from the nervous system, intestine, or gonad. These cell nonautonomous signaling pathways not only enhance lifespan and stress resilience but also limit age-related accumulation of protein aggregates that exacerbate age-associated diseases. Thus, findings from aging research were often key to providing new insights into cell nonautonomous regulation of stress responses and organismal proteostasis. In this review, we outline key discoveries made using *C. elegans* as a model system and highlight their contributions that led to our current understanding of inter-tissue communication in organismal proteostasis regulation. We furthermore highlight emerging concepts and discuss the translational relevance of conserved cell nonautonomous proteostasis regulation in mammals. We emphasize the importance of mammalian research to support the research done in *C. elegans,* with the future goal of developing potential therapeutic interventions targeting these inter-tissue proteostasis signaling pathways to combat aging.

## How *Caenorhabditis elegans* became a model system to study organismal proteostasis and aging

### The proteostasis network supervises proteome integrity and acts to orchestrate organismal aging

Maintenance of proteostasis is fundamental to cellular function and organismal health. The proteostasis network (PN) is comprised of molecular chaperones, organelle-specific stress response mechanisms such as the unfolded protein responses (UPRs), the heat shock response (HSR), and degradation pathways including the ubiquitin–proteasome system (UPS) and autophagy–lysosome pathways, which are discussed in more detail in other reviews of this topic collection. Collectively they ensure the correct folding, trafficking, and timely degradation of proteins. At the cellular level, these mechanisms buffer against protein misfolding and aggregation, ensuring proteome integrity. At an organismal level, the same mechanisms function across tissues and are communicated via intercellular stress signaling processes, allowing for a systemic coordination of PN components that is crucial for adapting to proteotoxic challenges during development, environmental stress, and aging. In particular, as organisms age, the PN has a key role in keeping signaling pathways, cellular maintenance, and stress response under control. If the PN starts to break down or decreases in ability to cope with cellular demands, this can contribute to the development and progression of age-associated diseases such as neurodegenerative diseases. Because of its importance in aging, proteostasis collapse is a key hallmark of aging: as an organism ages, the PN will start to break down and lead to the accumulation of misfolded proteins and associated pathologies.

**Fig. 1:**
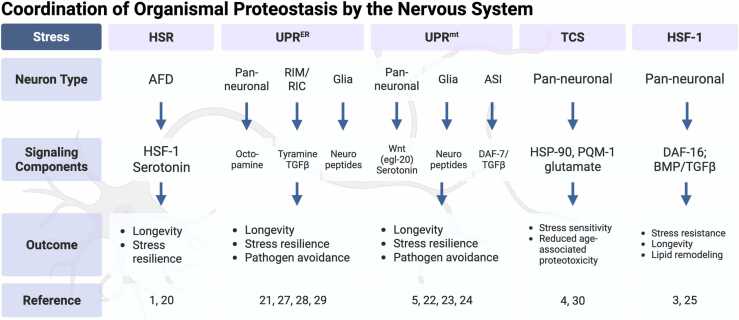
Coordination of organismal proteostasis by the nervous system. Representation of the major cell nonautonomous proteostasis signaling pathways that are coordinated by the nervous system, including the HSR, UPR^ER^, UPR^mt^, TCS, and HSF-1 mediated signaling. For each, the primary neuron type of signaling transmission, signaling components, and the outcome within *C. elegans* are shown, along with corresponding references. Created in BioRender. Van oosten-hawle, P. (2025) https://BioRender.com/6su85xk

Many of these intercellular stress-signaling pathways were first elucidated in the nematode *Caenorhabditis elegans*, which has become a powerhouse model system to study organismal proteostasis. Studies in *C. elegans* were the first to uncover the cell nonautonomous HSR coordinated by the nervous system as illustrated in Figure 1. Activation of amphid neurons with finger-like ciliated endings (AFD) thermosensory neurons regulates the HSF-1 mediated HSR between tissues.^1^ In addition to thermosensory neurons, chemosensory neurons also play a role in the cell nonautonomous induction of heat shock proteins via the G protein coupled receptor (GPCR) GTR-1.^2^ Moreover, chemosensory amphid wing “C” cells neurons were shown to regulate organismal proteostasis and longevity via microRNA-dependent signaling.^3^ Similarly, elevating HSF-1 expression exclusively within the nervous system extends lifespan, a process that requires intestinal DAF-16 and the bone morphogenic protein (BMP) pathway-associated intestinal small receptor, a type I TGF-β receptor normally involved in growth and body length regulation^4^ and reproductive aging^5^ Even altering chaperone expression levels in either neurons or the intestine is sufficient to induce a compensatory chaperone response in a distal tissue, a process termed Transcellular Chaperone Signaling (TCS).^6^ Likewise, the discovery of the cell nonautonomous mitochondrial UPR (UPR^mt^) and the UPR of the endoplasmic reticulum (UPR^ER^) in *C. elegans* demonstrated how stressed organelles send hormonal or neuropeptide signals across tissues in order to coordinate whole-body responses^7^, ^8^ (Figure 1). These seminal studies revealed that proteostasis can be governed by the nervous system as a central regulatory hub, with peripheral signaling tissues such as the intestine also contributing to the regulation of cell nonautonomous proteostasis, directly impacting aging processes within the organism. Deciphering the signaling pathways and networks that orchestrate proteostasis between cells is a prerequisite for harnessing these mechanisms in potential therapies designed to postpone age-associated protein folding disorders and tissue degeneration.

### The importance of *C. elegans*

The use of *C. elegans* as a model system has played an integral role in bringing these discoveries into light. As the insulin like growth factor receptor, IGF-1R (DAF-2 in *C. elegans*), and the insulin-like signaling (ILS) pathway were discovered in the early 1990s, it prepared the path for several breakthroughs to occur that aimed to understand how cell nonautonomous signaling can orchestrate the physiology of a multicellular organism and influence aging.^9^, ^10^ Landmark papers, using *C. elegans,* identified the role of the nervous system, in particular chemosensory neurons, as a hub for regulating aging and longevity.^11^, ^12^ The breakthroughs and discoveries that followed would have been far more challenging to find using larger model organisms, such as mice, and impossible within unicellular organisms, such as yeast. Because proteostasis pathways are highly conserved from yeast to man, *C. elegans* as a multicellular organism offers a unique tool and perspective to investigate aging and inter-tissue stress signaling that unicellular model systems, by definition, cannot offer. The short lifespan of *C. elegans* of only a few weeks combined with its transparent body aid in accurate visualization of proteostasis mechanisms throughout the life-course of the animal. Because of this, each impact on protein integrity or stress response pathways can be tracked throughout aging, and their influence on age-associated diseases can be further assessed. Individual components of the PN, including the chaperone network, or the “chaperome,”^13^ are highly conserved in *C. elegans* as well as humans. Recently the Proteostasis Consortium, a group of investigators who contributed fundamental insights on molecular and organismal processes of the PN, https://www.proteostasisconsortium.com/, has shared the human PN, a comprehensive list of components involved in translation, protein folding, and organelle-specific systems and components of the autophagy-lysosome pathways.^14^ Their efforts show and highlight that processes related to proteostasis are ancient and highly comparable across the animal kingdom. In fact, cell nonautonomous proteostasis signaling processes first observed in *C. elegans* have been recapitulated in mammals (discussed in Section 3), with the potential for translational therapeutic strategies aimed at neurodegenerative diseases, metabolic disorders, and general age-related deterioration.

Understanding how inter-tissue signaling responses influence organismal proteostasis and aging is still a relatively recent area of research. The fundamental discoveries made so far catalyze the development of future interventions that could modulate the required inter-tissue signal relay to benefit health span. In this review, we summarize key inter-tissue signaling mechanisms that orchestrate proteostasis both within and across tissues. We explore emerging trends on translational aspects of these cell nonautonomous signaling networks into potential therapeutic strategies for promoting organismal proteostasis and healthy aging.

## Tissue-hubs of transcellular stress signaling pathways that maintain organismal proteostasis

### Coordination of proteostasis across the organism by the nervous system

The discovery of the ILS pathway and definition of its components that regulate life span extension in *C. elegans*^9^, ^10^, ^15^ promoted further studies aimed at identifying cells in which *daf-2* acted to coordinate growth and aging.^12^ This led to the realization that *daf-2* functions cell nonautonomously to regulate lifespan within the organism and highlighted the importance of inter-tissue signaling as a new concept in the aging field.^12^ The DAF-2/ILS pathway depends on downstream components phosphatidylinositol 3-kinase homolog *age-1* and the HNF-3/forkhead transcription homolog DAF-16,^16^ with DAF-2 itself, as well as AGE-1, being sufficient to regulate lifespan when expressed in the nervous system only.^16^ *C. elegans* has a specific set of neurons, called amphid neurons, with ciliated projections that sense the environment and relay information, such as thermal challenges or food cues, to peripheral organs within the organism. Early experiments by the Kenyon lab using mosaic *daf-2* animals^12^ and ablation studies of specific olfactory and gustatory neurons^17^ showed that DAF-2 functions in a cell nonautonomous manner with gustatory neurons regulating longevity through the DAF-2/ILS pathway.^17^

This foundational research paved the way for subsequent studies investigating cell nonautonomous mechanisms regulating proteostasis, a key hallmark of aging. The ILS pathway modulates both HSF-1 and DAF-16 transcription factors,^18^, ^19^ with DAF-16 specifically governing the expression of small heat shock proteins (sHsps) but not other Hsp families.^18^ In contrast, the heat shock transcription factor HSF-1 orchestrates a broader chaperone network composed of Hsp90, Hsp70, Hsp60, and sHsps.^20^, ^21^ This extensive chaperone network is essential for lifespan extension mediated through the DAF-2/ILS pathway and dependent on HSF-1 activity in *C. elegans.*^21^ Following work then started to focus on how HSF-1 is regulated in a tissue-specific manner. Thermo-sensory AFD neurons^1^ and chemosensory neurons^2^ in *C. elegans* were identified for their role in regulating the cytosolic HSR mediated by HSF-1. Ablation of AFD neurons dampened the HSR and reduced expression of Hsp70 and Hsp90 as well as sHsp families in several tissues of the animal and reduced activation of HSF-1 by nuclear localization in germ cells.^1^, ^22^ However, whether this pathway acts in a *daf-2* dependent manner is not clear. Importantly, experiments using optogenetic stimulation of AFD neurons showed that HSF-1 and the HSR can be activated by AFD-downstream serotonergic neurons via serotonergic neurotransmission (Figure 1).^22^ Paradoxically, while the presence of functional AFD neurons is required to activate the HSR, inactivation of the same neurons can lead to chaperone upregulation that suppresses aggregation and toxicity caused by polyglutamine expansion proteins and mutant SOD-1-G39A.^23^ In line with this, knockdown of the chemosensory neuron expressed-GPCR *gtr-1*, alleviates amyloid beta (Aβ)-associated proteotoxicity in distal tissues while suppressing activation of the HSR.^2^ Together, this highlights that the ability of the animal to respond to acute heat stress can come at the expense to counteract chronic proteotoxicity at a cell nonautonomous level.

These studies all stress the importance that specific neuronal subtypes and the nervous system as a whole have on regulating a broader organismal response to stress and regulating stress response pathways such as the HSR.

Other studies have further explored how subcellular stress responses are regulated cell nonautonomously through neuronal signaling. For instance, neuronal expression of the activated form of XBP-1 (*xbp-1s*), the transcription factor involved in the IRE-1 branch of the UPR^ER^, activates the UPR^ER^ in the intestine.^8^ This inter-tissue signaling enhances stress resistance and lifespan extension and delays the age-related protein aggregation in peripheral tissues via octopaminergic neurotransmission.^24^ Similarly, mitochondrial stress specifically induced in neurons via knockdown of *cco-1* (complex IV of the ETC) induces the mitochondrial (mt) UPR in peripheral tissues.^7^ Follow-up studies identified Wnt signaling mediated through the Wnt receptor EGL-20 to be sufficient for cell nonautonomous induction of the UPR^mt^ in the intestine.^25^ In parallel to Wnt signaling, serotonergic neuronal signaling is also required for the cell nonautonomous UPR^mt^, albeit not essential.^25^ Thus, proteostasis as well as lifespan can be modified via neuronal activation of a stress response, requiring cell nonautonomous signaling often through neurotransmitters such as serotonin, but also other signaling cues in parallel. Another example, in addition to Wnt signaling, is the TGFβ signaling pathway, that was originally discovered to regulate dauer formation in *C. elegans.*^26^ The TGFβ pathway was demonstrated to regulate longevity through DAF-16, indicating that both the TGFβ and ILS pathways are tightly linked.^27^ In addition to DAF-16, neuronal HSF-1 has been shown to coordinate the BMP branch of the TGFβ signaling pathway across the “gut-brain-axis” to regulate longevity: It does so by reducing levels of the type I BMP receptor small, which in turn decreases peripheral signaling and modulates intestinal membrane trafficking.^28^ In addition to promoting longevity, neuronal HSF-1 overexpression induces fat remodeling in the intestine, characterized by reduced expression of a fat desaturase and a lipase along with shifting cell membrane composition towards an increase in phospholipids. This neuronal HSF-1-induced alteration of lipid metabolism in the gut is proposed to facilitate adaptation to higher temperatures.^29^ The neuron to gut signaling pathway responsible for modulating fat metabolism involves sensory neurons expressing TAX-2/TAX-4 and utilizes the TGFβ/BMP pathway for signaling across tissues^29^ (Figure 1). Interestingly, more recent work has shown that the nucleolar FIB-1-NOL-56-NOL-58 complex in somatic cells can influence TFGβ signaling in neurons, which modulates *C. elegans’* response to chronic proteotoxicity by regulating proteasome activity at an organismal level.^30^

Further research in the field has increasingly focused on pinpointing the specific neurons essential for activating cell nonautonomous stress responses: amphid neurons single ciliated ending sensory neurons are particularly relevant for the cell nonautonomous UPR^mt^, as they produce DAF-7, a TGFβ-like molecule that is secreted and targets the DAF-1/TGFβ receptors to orchestrate the cell nonautonomous UPR^mt^ and extend lifespan^31^ (Figure 1). Other specific neurons in *C. elegans*, such as the Ring motor neuron (RIM) Ring interneuron (RIC) interneurons, are involved in the cell nonautonomous activation of the UPR^ER^, particularly in the intestine, which results in lifespan extension. The biogenic amine, tyramine, which is synthesized in the RIM and RIC neurons, is required for this cell nonautonomous activation of the UPR^ER^, likely by binding to the octopamine receptor OCTR-1 in the intestine^32^ (Figure 1). Activation of *xbp-1s* in the same interneurons also occurs in response to the pathogen-associated odorant 1-undecene, which triggers a cell nonautonomous UPR^ER^ to the intestines.^33^ However, while this odorant-induced UPR^ER^ activation requires the RIM-RIC interneurons, it depends on TGFβ-signaling instead of neurotransmitters.^33^ Importantly, the response is associated with behavioral consequences such as pathogen avoidance alongside molecular defensive mechanisms that induce the immune response.^33^ Surprisingly, expression of XBP-1s not only functions in neuronal cells but is sufficient in glia, neuronal support cells, to induce the UPR^ER^ in peripheral tissues, and is dependent on neuropeptides, rather than neurotransmitters.^34^ Thus, overall, sensory neurons not only perceive the environment but play a key role in the regulation of lifespan and coordination of proteostasis and molecular stress responses across tissues.

It is conceivable that cell nonautonomous proteostasis is not only regulated via stress response pathways that have counterparts at the cell autonomous levels, such as the HSR, UPR^mt^, and UPR^ER^, but that there might be other pathways that regulate proteostasis components at an organismal level only. One such pathway is TCS that is triggered by alterations in expression levels of Hsp90, a key molecular chaperone involved in the regulation of a plethora of cellular signaling processes through its interactions with kinase proteins and transcription factors.^6^ In cellular proteostasis, knockdown of Hsp90 can induce the HSF-1-mediated HSR, whereas overexpression can block it,^20^ thus linking its regulation to the cytosolic HSR. Using *C. elegans,* it was shown that tissue-specific overexpression of Hsp90 in the neurons was sufficient to block the HSR in peripheral tissues, leading to reduced stress resilience in response to heat stress. Despite this, the increased levels of Hsp90 in the neurons suppressed age-associated chronic stress and amyloid beta (Aβ) protein aggregation toxicity in the neurons themselves as well as in peripheral tissues such as muscle.^6^, ^35^ This mechanism depends on glutamatergic neurotransmission and activation of both stress-responsive transcription factors PQM-1 and PHA-4 to mediate the response cell nonautonomously^35^ (Fig. 1, Fig. 4), albeit the precise sensory neurons coordinating this response remain to be determined. These observations further highlight the complexity of signaling mechanisms mediating a nonautonomous response and emphasizes the importance of sensory perception being able to drive not only behavioral changes but also proteostastic consequences at the organismal level.

### Coordination of cell nonautonomous proteostasis by the reproductive system

**Fig. 2:**
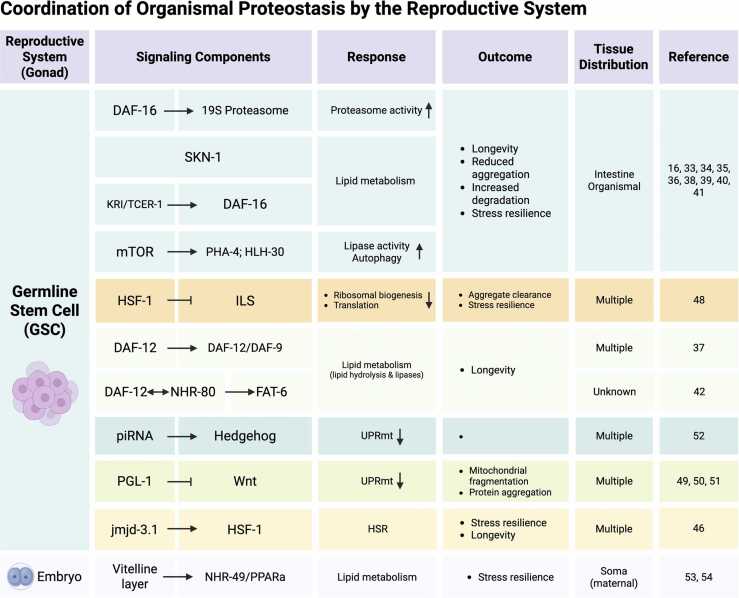
Coordination of organismal proteostasis by the reproductive system. Major organismal proteostasis signaling pathways that coordinated by the reproductive system, with primarily germline stem cells (GSCs) and embryos being emphasized. For each pathway the signaling components are shown, followed by organismal responses and outcomes, tissue distribution within *C. elegans* and cited articles referring to each observation. Created in BioRender. Van oosten-hawle, P. (2025) https://BioRender.com/h2x5rxm

When proliferating germ cells are removed, *C. elegans* live 60% longer and are resistant to a variety of environmental challenges.^36^, ^37^ In contrast, removal of the entire reproductive system, which includes the gonad and germ cells has no effect on longevity.^36^ This demonstrates that germline stem cells (GSCs) regulate the longevity of the soma in an animal.^37^, ^38^ Germline removal can be accomplished by genetic or laser ablation or by mutations in genes that are required for germ cell proliferation, such as the N-glycosylated transmembrane protein homolog of Notch, *glp-1* in *C. elegans*. *glp-1 (ts)* mutants cause premature meiosis during germ cell differentiation and lead to germline-less adults that are long-lived.^37^ Several pathways have been suggested to respond to lifespan-extending signals originating from the GSC to somatic cells, as summarized in Figure 2. DAF-16 integrates signals from different pathways to modulate aging and longevity, which includes the Target of Rapamycin pathway,^39^ 5' adenosine monophosphate-activated protein kinase signaling,^40^ the c-Jun N-terminal kinase pathway,^41^ Notch signaling,^42^ and ROS signaling.^43^ In addition to that, it can also be activated through the intestinal ankyrin-repeat protein KRI-1 and the transcription elongation factor TCER-1.^44^ Moreover, transcription factors HSF-1, SKN-1, and PHA-4, in addition to DAF-16, were shown to regulate germline-mediated longevity, often involving lipid metabolism as a response.^18^, ^45^, ^46^, ^47^, ^48^ Other pathways depend on a lipophilic hormone signaling pathway requiring the steroid hormone receptor DAF-12 (homolog of vitamin D receptor), and the cytochrome P450 DAF-9, which is thought to make a lipophilic ligand for DAF-12.^44^, ^49^ A link with fat metabolism was later on established with the observation that GSC arrest could modulate lipid hydrolysis and fat metabolism in *C. elegans* and extend longevity.^38^ Follow-up work showed that the involvement of the HLH-30, PHA-4, DAF-16 transcription factors, as well as the nuclear receptors NHR-49 and DAF-12^45^, ^48^, ^50^, ^51^, ^52^ all link the pro-longevity effect of germline-lacking worms with lipid metabolism. In addition, the nuclear hormone receptor NHR-80/HNF4 is upregulated as a consequence of GSC depletion and controls the transcription of the stearoyl-CoA-desaturase *fat-6*, which desaturates stearic acid to oleic acid to promote longevity in a DAF-12 dependent manner.^52^ In summary, activation of these factors upregulates genes involved in fatty acid β-oxidation, lipophagy, and fatty acid desaturation, which are all required for lifespan extension (Figure 2). However, *glp-1* mutants also show increased lipid storage, which is a paradox, as many of the aforementioned factors are involved in lipid mobilization rather than storage.^53^ This can, however, be explained by the accumulation of yolk lipids that are unused due to lack of oocytes in mutants lacking a germline.^48^

How might the germ cells communicate with the soma to impinge on activation of stress responses as animals age to maintain organismal proteostasis for longer? A key concept began to emerge with the observation that *C. elegans* gradually lose the ability to maintain metastable proteins in a functional state and to mount an effective stress response. This led to the proposition that the collapse of proteostasis occurs by day 3 of adulthood.^54^ Interestingly, removal of the germline enhances the proteostasis capacity of somatic cells. This was initially attributed to increased clearance mechanisms of age- or disease-associated protein aggregates mediated through the proteasome and autophagy-lysosome systems.^46^ In particular, germline-less animals have increased proteasome activity due to higher expression of the 19S proteasome subunit *rpn-6*.1 that is regulated by DAF-16.^46^ The mechanism itself may be conserved in humans, as one of the DAF-16 homologs, FOXO4, is necessary for increased proteasome activity and RPN-6/PSMD11 expression in human embryonic stem cells.^55^ The enhanced autophagy activity of germline-deficient animals is also tied to increased lipolysis and lipase (*lipl-4*) activity, which is controlled by mTOR and both PHA-4 and DAF-16 transcription factors to drive expression of several autophagy genes.^45^, ^56^ Subsequent studies added the conserved transcription factor HLH-30/TFEB to this regulatory network, demonstrating its role in promoting autophagy and longevity in germline-less animal.^51^

Signals from the reproductive system clearly shape organismal metabolism with consequences for stress resistance and lifespan. But how do they drive the decline of cellular proteostasis occurring in early adulthood? This decline was identified to occur within a narrow four-hour window at the onset of egg-laying, during which animals lose the ability to mount an effective HSF-1-mediated heat stress response.^57^, ^58^ This loss of the HSR is driven by GSC-derived signals that block the expression of H3K27me3 demethylase *jmjd-3.1*, leading to increased H3K27me3 marks at stress gene loci, widespread shutdown of stress responses, and a reset of proteostasis capacity in somatic cells.^57^ Consistent with the established role of the germline in lifespan regulation, the primary requirement for HSF-1 in germline development is dictated by ILS activity.^59^ A recent paper showed that given the germline being the primary site for protein synthesis in reproductive animals, HSF-1 dependent chaperone expression must be carefully balanced with the ILS pathway to optimize protein folding capacity for gametogenesis.^60^ When HSF-1 activity is lost, reduced ILS enhances stress resilience of the germline by decreasing ribosomal biogenesis and translation rates in gametes. This downregulation emphasizes protein folding and clearance pathways, allowing effective management of misfolded proteins during stress and thereby enhancing stress resilience.^60^

In addition to the cytosolic HSR, other stress response pathways, including the UPR^ER^ and UPR^mt^, are also regulated by GSCs to influence proteostasis and stress response activation in a cell nonautonomous manner. For instance, aggregation of the RNA-binding protein PGL-1 in germ cells not only disrupts mitochondrial integrity within the germline itself but also impairs mitochondrial function in somatic tissues through Wnt signaling. This impairment triggers inter-tissue activation of the UPR^mt^, mitochondrial fragmentation, and aggregation of disease-related proteins in various tissues, including the nervous system, muscle, and intestine.^61^ An intact reproductive system appears essential for UPR^mt^ induction in somatic tissues; specifically, sperm cells are required to activate the somatic UPR^mt^.^62^ Importantly, recent evidence suggests the germline plays a central coordinating role for UPR^mt^ activation across tissues: Germline mitochondria function downstream of neuronal mitokine signaling and Wnt, yet upstream of lipid metabolic pathways to orchestrate cell nonautonomous mitochondrial stress responses.^63^ Moreover, the germline produces Hedgehog-like ligands *wrt-5* and *wrt-6*, that are targeted and suppressed by piRNAs. This event then signals to somatic tissues (primarily the intestine), suppressing the UPR^mt^ and resulting in a progressive decline in UPR^mt^ responses.^64^

Strikingly, it is not only GSCs that are essential for coordinating organismal proteostasis in maternal somatic tissues. The health and integrity of embryos developing within the uterus can also initiate transcellular signaling pathways that enhance stress resilience in the maternal somatic cells.^65^, ^66^ Specifically, recent findings show that a damaged extracellular coat surrounding the developing embryo (the vitelline layer) activates a signaling cascade that increases stress resistance in the mother through mechanisms dependent on the transcription factors DAF-16 and HSF-1 in somatic tissues.^65^ In addition, the same damage to the embryonic vitelline layer also alters lipid content of the mother by increasing intestinal fat storage, driven in part by the NHR-49/PPARa transcription factors.^66^ This also benefits the maternal HSR and stress resilience by influencing HSF-1 activity, although the molecular mechanism by which NHR-49 could regulate HSF-1 activity remains to be determined.

### Longevity mechanisms in the intestine: Lipid metabolism, chaperones, and the microbiome as regulators of proteostasis and longevity

#### The intestine as a cell non autonomous aging hub

**Fig. 3:**
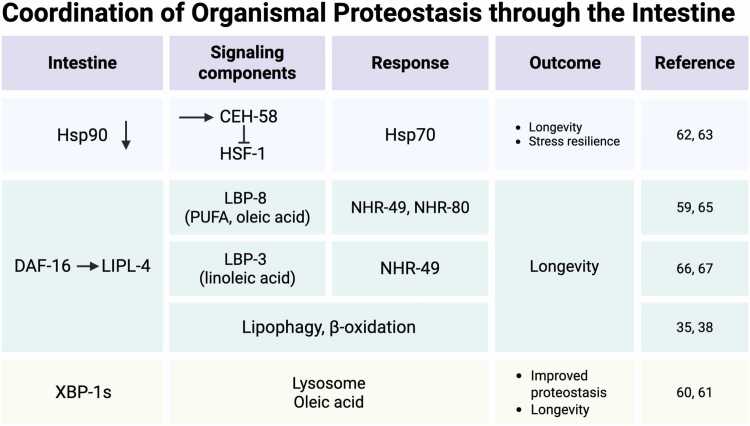
Coordination of organismal proteostasis through the intestine. Major organismal proteostasis signaling pathways coordinated by the intestine are shown. For each pathway, the involved signaling components, organismal responses, and outcomes within *C. elegans* are demonstrated as evidenced by corresponding references. Created in BioRender. Van oosten-hawle, P. (2025) https://BioRender.com/cg0gqcn

In *C. elegans*, the intestine functions as a major endocrine center that governs both aging and proteostasis in distant tissues. Early work on the ILS pathway revealed its importance when DAF-16 expressed in the neurons of *daf-2, daf-16* mutants increased lifespan by 20%, whereas intestinal expression of DAF-16 restored 60% of the *daf-2* longevity phenotype.^11^ This identified the gut as a crucial hub in regulating lifespan and aging.^11^ Subsequent studies showed that DAF-16 regulates downstream genes that affect longevity in a cell-autonomous manner but that also delays aging nonautonomously by inducing target genes and potential secreted factors that activate parallel longevity pathways in other tissues.^27^ Importantly, intestinal DAF-16 not only prolongs lifespan but also suppresses amyloid protein aggregation in the muscle, demonstrating the organismal impact of the gut on proteostasis.^27^, ^67^ Consistent with these endocrine effects, age-associated death in *C. elegans* was later reported to be “initiated” in the gut.^68^ Beyond signaling, it is important to remember that the intestine is also the major site of fat storage in *C. elegans* and therefore acts as the adipose tissue of the organism. Indeed, *C. elegans* has emerged as an important model linking metabolism, diet, and aging. Initial studies connecting longevity pathways to lipid metabolism uncovered key mechanistic insights. The importance of intestinal DAF-16 in regulating longevity was due to its role in driving expression of lipid metabolic genes, including fatty acid (FA) desaturases, lipases, and lipid transport proteins Figure 3.^27^ For example, *daf-16* dependent upregulation of the Δ9 desaturase FAT-7 increases monounsaturated fatty acids (MUFAs), including oleic acid,^69^ and dietary supplementation with oleic acid alone is sufficient to extend lifespan.^70^ The lysosomal acid lipase *lipl-4* is another DAF-16 target in the intestine, and its overexpression was shown to promote longevity through increased lipophagy and mitochondrial β-oxidation.^45^, ^71^ Moreover, DAF-16 cooperates with the ER stress regulator XBP-1 to control UPR^ER^ induction in the intestine to regulate lifespan Figure 3.^72^ Reciprocally, XBP-1s, when expressed in neurons induces gut lipophagy, reduces fat storage, and elevates oleic acid levels, linking neuronal UPR^ER^ signaling to peripheral lipid metabolism.^73^

**Fig. 4:**
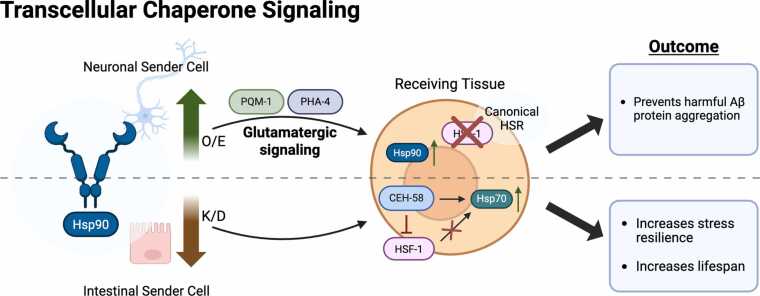
Schematic representation of transcellular chaperone signaling. Hsp90 overexpression in the nervous system blocks HSF-1 and the HSR by activating glutamatergic signaling and the transcription factor PQM-1, as well as PHA-4. Hsp90 knockdown in the intestine results in the induction of Hsp70 at a cell nonautonomous level through the homeodomain transcription factor CEH-58, which antagonizes HSF-1 activity and the canonical HSR. Created in BioRender. Van oosten-hawle, P. (2025) https://BioRender.com/vphzppe

Beyond lipids and hormones, the gut also relays proteostatic stress via chaperones. Knocking down *hsp-90* specifically in the gut triggers a cell nonautonomous response: heat-inducible *hsp-70* becomes upregulated in distal tissues even at the permissive temperature, boosting both lifespan and thermotolerance^74^ (Figure 3 and Figure 4). This non-canonical *hsp-70* induction bypasses HSF-1 and instead relies on the homeodomain transcription factor CEH-58, which antagonizes HSF-1 activity (summarized in Figure 4). Although under acute heat stress, the classical HSF-1–mediated HSR prevails in control animals; the chronic intestine-specific *hsp-90* depletion engages a TCS pathway that constitutively elevates Hsp70 elsewhere while actively suppressing HSF-1-driven Hsp70 transcription^74^ (Figure 4). The physiological importance of this observation is follow-on work showing that Hsp90 itself is degraded by the lysosome specifically in the intestine after a thermal stress.^75^ Upon heat shock, the CUL-6 ubiquitin ligase, which is expressed in the intestine, targets HSP-90 for ubiquitylation and directs it to lysosome-related organelles where it is degraded and which in turn promotes organismal survival.^75^ Interestingly, *cul-6* is also a gene upregulated by the intracellular pathogen response, a stress resistance pathway previously identified to promote thermotolerance in *C. elegans.*^76^ This mechanism underscores how multicellular organisms have evolved layered, tissue-specific signaling networks to coordinate proteostasis. Although lipid metabolism has not been directly connected with this pathway so far, the involvement of gut-derived lipases in TCS signaling^74^ and the requirement for lysosomal degradation^75^ suggest that additional studies are needed to clarify this potential connection.

#### Lipids as signaling mediators

In what ways do lipids promote longevity and coordinate organismal proteostasis across different tissues? Firstly, they are fundamental elements of all cellular membranes, maintaining the separating barrier of the cell itself from its immediate extracellular environment. Secondly, lipids can assemble into caveolae and ER-mitochondria contact sites (MAMs), which act as signaling hubs for proteostasis regulation in worms and are linked to Alzheimer’s disease.^77^ Lipid droplets additionally function as proteostasis buffers that transiently sequester misfolded proteins to aid clearance.^78^ Thirdly, they can act as signaling molecules, either by influencing membrane fluidity or by activating signal transduction pathways, intracellularly, or inter-cellularly, through binding to nuclear hormone receptors, GPCRs, and other lipid-binding proteins. Free FAs require lipid binding proteins, which facilitate their trafficking and signaling effects.^79^ For example, the *C. elegans* lipid binding protein LBP-8 is upregulated in long-lived strains that overexpress *lipl-4*. It shuttles poly-unsaturated fatty acids as well as oleic acid to the nuclear receptor complex NHR-49-NHR-80, to drive pro-longevity transcription.^71^, ^80^ Similarly, LBP-3 was recently shown to be important for inter-tissue transport of dihomo-γ-linolenic acid from the intestine to the nuclear hormone receptor NHR-49 in neurons to extend lifespan.^81^ NHR-49 itself senses lipid depletion by being sequestered on endocytic vesicles through RAB-11.1 via geranylgeranyl conjugation.^82^ This results in reduced nuclear activity of NHR-49 in times when lipids are present sufficiently. When lipid stores are depleted, NHR-49 is released and induces the expression of β-oxidation and peroxisome genes.^82^ This demonstrates that nuclear receptor activity can be tuned to the metabolic state of the cell and the organism. How can MUFAs such as oleic acid extend lifespan and benefit organismal proteostasis? Clues for a molecular mechanism came from a recent study showing that MUFAs remodel the cellular architecture in *C. elegans*: they boost lipid droplets and peroxisome abundance. At the same time, they reduce peroxidation-prone ether lipids, thereby optimizing membrane composition and enhancing longevity.^83^

Feeding *C. elegans* with different diets can change metabolism and influence proteostasis in other tissues. For example, when a *C. elegans* Parkinson’s disease model, expressing α-syn in muscle cells, is fed the probiotic *Bacillus subtilis*, the host sphingolipid metabolism is remodeled, which consequently reverses and inhibits α-syn aggregation. Although specific sphingolipid metabolism genes were identified in the protective effect, the exact mechanisms on how this impacts α-syn aggregation remains to be determined.^84^ More broadly, the human microbiome has long been implicated in age-related diseases.^85^, ^86^ A recent study now sheds more light on this aspect by using a comprehensive screen of 229 bacterial isolates form the Human Microbiome Project and investigating their effect on different age-associated diseases in the host. Among these bacterial isolates, *Prevotella corporis* was identified as one of the strongest contenders that benefitted host proteostasis by reducing aggregation and proteotoxicity of polyQ, Aβ_(1−42)_ and α-synuclein in peripheral tissues.^87^ Albeit the precise mechanism remains unclear, the protective effect of *P. corporis* depends on induction of the HSR.^87^

## Emerging trends and future directions

### Other peripheral tissues involved in longevity regulation and proteostasis: Is there a hierarchy?

In humans, cell nonautonomous signal transduction often relies heavily on the nervous system. When we experience certain sensations originating from physiological signals, such as hunger from signals originating in the stomach or the sensation of touch, these signals typically first travel to the brain or brainstem. From there, a coordinated response is sent back to the originating tissues, instructing them how to react appropriately. From this viewpoint, the nervous system clearly acts as a central integrator of these physiological signals orchestrating responses across tissues. Yet our bodies utilize alternative pathways to communicate across organs, such as endocrine signals traveling through the lymph and bloodstream. For example, within the nervous system itself, signaling mechanisms differ; some rely on neurotransmitters, while others utilize alternative signaling pathways originating from peripheral organs. At the same time, given their sensory nature, neurons are uniquely positioned to translate external signals from the environment to systemic cues through neuronal networks. By contrast, organs like the pancreas, stomach, muscle, or intestines, on the other hand, sense their local environments and directly transmit signals to distant tissues, including the brain. Each organ or tissue contributes uniquely depending on context and the variety of internal or external stresses organisms continuously face. For example, human skeletal muscle releases myokines into the bloodstream during exercise that influence the brain, liver, and adipose tissue.^88^, ^89^ Myokines, including the interleukins IL-6, IL-7, and IL-15 regulate metabolic processes such as hepatic glucose production,^90^ while the myokine FNDC5/irisin can rescue synaptic plasticity and memory function in mouse models of Alzheimer’s disease.^91^ More recent studies from Drosophila using metabolic labeling and proteomics have identified 51 muscle secreted proteins in the fly’s head, highlighting a substantial pool of factors mediating muscle-to-brain communication that will require further investigation in the future.^92^ Another example is the hypodermis that actively signals to neighboring and distant tissues, influencing fat metabolism inflammation in humans.^93^ A recent study using *C. elegans,* the hypodermis was shown to cell nonautonomously regulate neuronal activity and memory formation via a DAF-2-dependent modulation of the diffusible Notch ligand OSM-11, which improves memory and slows cognitive decline.^94^

Whether or not one tissue is hierarchically above another often depends on the origin of the stress, the specific signals involved, and the developmental stage or age of the organism. For example, the germline and, in particular, germline mitochondria take on a “mediator role” communicating between the nervous system and the intestine in *C. elegans*. In this model proposed by Shen et al (2024), the germline receives a mitokine signal from stressed neurons and emit a secondary signal to the intestine to regulate the UPR^mt^.^63^ This signal exchanged between the neurons and the germline was suggested to involve components of the Wnt signaling pathway^25^ which can directly regulate oocyte mitochondria and mitochondrial DNA levels. Other implicated signaling pathways involved in this process were shown to rely on serotonin signaling, which is known to improve oocyte quality.^95^ Specifically, during external heat stress conditions, 5-HT, the precursor molecule of serotonin, is released from maternal neurons to accelerate HSF-1-dependent transcriptional protective responses, such as the expression of heat shock proteins. Such a mechanism ensures the viability and stress resilience of these future offspring.^96^ Further emphasizing the significance of chromatin dynamics, it has been demonstrated that the duration of maternal heat stress can lead to a heritable epigenetic memory passed on to subsequent generations. Interestingly, HSF-1 can erase transcriptional memory and paradoxically recruits the germline machinery to establish this stress-associated memory.^97^ The developmental timing of stress exposure is also critical, as the germline, at the onset of reproduction, suppresses expression of the histone demethylase *jmjd-3.1* which, in somatic cells, leads to reduced ability of stress-responsive transcription factors to bind to stress response genes, which impairs the ability of the organism to induce an adequate protective stress response, leading to proteostasis collapse during reproductive onset.^57^ However, whether this is regulated through input of the nervous system or independently and solely through the germline remains to be determined. Oxidative stress early in life can also influence organismal longevity through HSF-1-dependent alterations in histone landscapes.^98^ This protective effect involves the inactivation of the redox-sensitive COMPASS complex, responsible for depositing the histone modification H3K4me3, and is driven by enhanced heat-shock-independent activity of HSF-1. Such alterations lead to decreased mitochondrial beta-oxidation and an increase in protective MUFAs such as oleic acid, which have implications for age-associated conditions like Alzheimer’s disease.^98^

In closing, these findings underscore the complexity of inter-tissue communication and highlight the dynamic and context-dependent roles played by different tissues and signaling pathways in maintaining organismal health and resilience. Each organ or tissue therefore bears its own responsibility for maintaining proteostasis through self-governance while remaining in dialog with the rest of the body. Such a coordination suggests an *ontocratic* model: Rather than the nervous system ruling in a top-down *autocratic* hierarchy, each organ is self-regulating and stress condition-aware, yet also aligned with the interconnected unity of the organism as a whole. Particularly in the case of *C. elegans*, the interplay between the nervous system, germline, intestine, and other tissues demonstrates how carefully orchestrated signaling cascades not only maintain homeostasis but also enable adaptive responses to environmental stresses. Ultimately, unraveling these interactions at the cellular and molecular levels may provide valuable insights into mechanisms of aging and disease, potentially opening new avenues for therapeutic interventions in humans.

### Is mammalian proteostasis regulated in a cell nonautonomous manner with consequences for aging?

The studies in the invertebrate *C. elegans* have provided new insights into cell nonautonomous proteostasis mechanisms and their relevance for aging and the progression of age-associated diseases. Are these studies relevant for mammals and, by extension, for humans? Few studies have explored the effects of cell nonautonomous proteostasis signaling and aging in mammalian model systems to date. This is due to obvious reasons, as mammals require an increased amount of time and resources to conduct such experiments. In this section we will discuss a few individual studies which suggest that regulation of organismal proteostasis is a conserved phenomenon. For example, similar to *C. elegans*, reduced insulin and growth hormone signaling also increases mouse lifespan^99^, ^100^ and was shown to protect mice from Aβ toxicity.^101^, ^102^, ^103^ In a similar manner, genetic studies conducted with human centenarians support a role for insulin signaling in human lifespan, as polymorphisms in the gene region of insulin (INS) and insulin growth factor 2 (IGF2) were significantly associated with lifespan extension.^104^

Further evidence that proteostasis-regulating transcellular stress signaling responses are a conserved phenomenon that is not only restricted to invertebrates was shown for the UPR^ER^ in a mouse model in which expression of XBP-1s in the pro-opiomelanocortin (POMC) neurons, which respond to insulin and leptin, activated XBP-1 and hence the UPR^ER^ in hepatic cells within the liver.^74^ This improved insulin sensitivity and increased energy expenditure in these mice, protecting them from diet-induced obesity.^105^ Similarly, serotonin, which was identified as a neuronal signaling messenger that activates the HSR in peripheral tissues within *C. elegans* and protects them from harmful protein aggregation^1^, ^22^, also mitigates age-associated proteotoxicity in mouse models.^75^ For example, citalopram, a selective serotonin reuptake inhibitor, which elevates serotonin levels, protects mice expressing polyglutamine-containing disease protein Ataxin 3, which causes Machado Joseph Disease in humans.^106^ Another example is the selective serotonin reuptake inhibitor fluoxetine, that protects middle-aged Alzheimer’s disease mice from neuronal loss.^107^

A large-scale transcriptional profiling study of aging human brains identified a core set of 16 chaperones, including Hsp70s and Hsp90, that decrease in aged individuals as well as in Alzheimer’s disease patients’ brains. Could elevating the expression of these chaperones in the brain mitigate age-associated decline? Clues that this is indeed the case stem from a previous study investigating the effects of intranasally administered recombinant human Hsp70 in mice.^108^ The Hsp70-treated mice showed a 10% lifespan extension, combined with improved memory during old age. Interestingly, they also demonstrated higher neuronal density in the cortex and hippocampus as well as accumulation of proteasomal subunits in the cerebral cortex.^77^ Thus, increased Hsp70 levels in mouse brains may promote proteasomal activity and extend lifespan cell nonautonomously in mammals; however, the exact mechanism remains to be determined. Overall, these studies clearly indicate a conservation for cell nonautonomous stress signaling processes in mammals, even if the level of known molecular detail is much lower compared to studies in *C. elegans*. Invertebrate research will likely continue to pioneer discoveries in this area in the foreseeable future, but the identified inter-tissue proteostasis signaling networks require the mammalian counterpart to assess and develop future interventions with potentially exciting new opportunities to improve human health span.

## CRediT authorship contribution statement

**Loren Cocciolone:** Writing – review & editing, Visualization. **Akhil Souparnika:** Writing – review & editing. **Valeria Uvarova:** Writing – review & editing. **Katie Kessler:** Writing – review & editing. **Patricija van Oosten-Hawle:** Writing – review & editing, Writing – original draft, Visualization, Funding acquisition, Conceptualization.

## Funding and support

This work was supported by NIH R01 AG082970 and start-up funds from UNC Charlotte to P.V.O.-H.

## Declaration of Competing Interest

The authors declare that they have no known competing financial interests or personal relationships that could have appeared to influence the work reported in this paper.

## Data Availability

No data was used for the research described in the article.
